# Supplementary material for: Biological Prognostic Value of miR-155 for Survival Outcome in Head and Neck Squamous Cell Carcinomas: Systematic Review, Meta-Analysis and Trial Sequential Analysis
Source: Biology (Basel). 2022 Apr 24;11(5):651. doi: 10.3390/biology11050651 (PMC9138061; doi:10.3390/biology11050651)
Supplement: Supplementary file 1 [file biology-11-00651-s001.zip › supplementary file S1.pdf]

Complete overview of the search methodology

Databases	K words, search details.	records
**PubMed**	Search: mir-155 AND HNSCC Sort by: Most Recent<br>"mir-155"[All Fields] AND ("hnsccs"[All Fields] OR "squamous cell carcinoma of head and neck"[MeSH Terms] OR ("squamous"[All Fields] AND "cell"[All Fields] AND "carcinoma"[All Fields] AND "head"[All Fields] AND "neck"[All Fields]) OR "squamous cell carcinoma of head and neck"[All Fields] OR "hnscc"[All Fields])<br>Translations<br>HNSCC: "hnsccs"[All Fields] OR "squamous cell carcinoma of head and neck"[MeSH Terms] OR ("squamous"[All Fields] AND "cell"[All Fields] AND "carcinoma"[All Fields] AND "head"[All Fields] AND "neck"[All Fields]) OR "squamous cell carcinoma of head and neck"[All Fields] OR "hnscc"[All Fields]	26
**PubMed**	Search: Microrna AND HNSCC AND prognosis Sort by: Most Recent<br>("microrna s"[All Fields] OR "micrornas"[MeSH Terms] OR "micrornas"[All Fields] OR "microrna"[All Fields]) AND ("hnsccs"[All Fields] OR "squamous cell carcinoma of head and neck"[MeSH Terms] OR ("squamous"[All Fields] AND "cell"[All Fields] AND "carcinoma"[All Fields] AND "head"[All Fields] AND "neck"[All Fields]) OR "squamous cell carcinoma of head and neck"[All Fields] OR "hnscc"[All Fields]) AND ("prognosis"[MeSH Terms] OR "prognosis"[All Fields] OR "prognoses"[All Fields])<br>Translations<br>Microrna: "microrna's"[All Fields] OR "micrornas"[MeSH Terms] OR "micrornas"[All Fields] OR "microrna"[All Fields]<br>HNSCC: "hnsccs"[All Fields] OR "squamous cell carcinoma of head and neck"[MeSH Terms] OR ("squamous"[All Fields] AND "cell"[All Fields] AND "carcinoma"[All Fields] AND "head"[All Fields] AND "neck"[All Fields]) OR "squamous cell carcinoma of head and neck"[All Fields] OR "hnscc"[All Fields]<br>prognosis: "prognosis"[MeSH Terms] OR "prognosis"[All Fields] OR "prognoses"[All Fields]	408
**PubMed**	Search: miR-155 AND OSCC Sort by: Most Recent<br>"miR-155"[All Fields] AND "OSCC"[All Fields]	21
**PubMed**	Search: LSCC AND miR-155 Sort by: Most Recent<br>"LSCC"[All Fields] AND "miR-155"[All Fields]	4
**SCOPUS**	TITLE-ABS-KEY (mir-155 AND hnscc)	11
**SCOPUS**	TITLE-ABS-KEY (microrna AND hnscc AND prognosis)	215
**SCOPUS**	TITLE-ABS-KEY (mir-155 AND scc)	25
**SCOPUS**	TITLE-ABS-KEY (LSCC AND miR-155)	5
**Cochrane library**	miR-155	50
**Records total**		765
**Number of records after removing duplicates**		549
