# Supplementary material for: Biological Prognostic Value of miR-155 for Survival Outcome in Head and Neck Squamous Cell Carcinomas: Systematic Review, Meta-Analysis and Trial Sequential Analysis
Source: Biology (Basel). 2022 Apr 24;11(5):651. doi: 10.3390/biology11050651 (PMC9138061; doi:10.3390/biology11050651)
Supplement: Supplementary file 1 [file biology-11-00651-s001.zip › supplementary file S2.pdf]

Quality Assessment Tool for Observational Cohort and Cross-Sectional Studies

- Study Quality Assessment Tools

Reference (first author and year of study	Was the research question or objective in this paper clearly stated?	Was the study population clearly specified and defined?	Was the participation rate of eligible persons at least 50%?	Were all the subjects selected or recruited from the same or similar populations (including the same time period)? Were inclusion and exclusion criteria for being in the study prespecified and applied uniformly to all participants?	Was a sample size justification, power description, or variance and effect estimates provided?	For the analyses in this paper, were the exposure(s) of interest measured prior to the outcome(s) being measured?	Was the timeframe sufficient so that one could reasonably expect to see an association between exposure and outcome if it existed?	For exposures that can vary in amount or level, did the study examine different levels of the exposure as related to the outcome (e.g., categories of exposure, or exposure measured as continuous variable)?	Were the exposure measures (independent variables) clearly defined, valid, reliable, and implemented consistently across all study participants?	Was the exposure(s) assessed more than once over time?	Were the outcome measures (dependent variables) clearly defined, valid, reliable, and implemented consistently across all study participants?	Were the outcome assessors blinded to the exposure status of participants?	Was loss to follow-up after baseline 20% or less?	Were key potential confounding variables measured and adjusted statistically for their impact on the relationship between exposure(s) and outcome(s)?
**Jakob (2019) [1]**	y	y	y	y	y	y	y	NA	NA	NA	y	NR	y	y
**Hess (2017) [2]**	y	n	y	y	y	y	y	NA	NA	NA	y	NR	y	n
**Zhao (2018) [3]**	y	n	y	y	y	y	y	NA	NA	NA	y	NR	y	n
**Baba (2016) [4]**	y	y	y	y	y	y	n	NA	NA	NA	y	NR	y	n
**Shi (2015)[5]**	y	y	y	y	n	y	n	NA	NA	NA	y	NR	y	n
**Kim (2018)[6]**	y	y	y	y	y	y	y	NA	NA	NA	y	NR	y	n

**Bersani (2018) [7]**	y	y	y	y	y	y	n	NA	NA	NA	y	NR	y	n
**Wu (2020) [8]**	y	y	y	y	y	y	y	NA	NA	NA	y	NR	y	y

N, no; NA, not applicable; U, unclear; Y, yes.CD, cannot determine; NR, not reported.

1. Jakob, M.; Mattes, L.M.; Küffer, S.; Unger, K.; Hess, J.; Bertlich, M.; Haubner, F.; Ihler, F.; Canis, M.; Weiss, B.G., et al. MicroRNA expression patterns in oral squamous cell carcinoma: hsa-mir-99b-3p and hsa-mir-100-5p as novel prognostic markers for oral cancer. Head and Neck 2019, 41, 3499-3515, doi:10.1002/hed.25866.
2. Hess, A.K.; Müer, A.; Mairinger, F.D.; Weichert, W.; Stenzinger, A.; Hummel, M.; Budach, V.; Tinhofer, I. MiR-200b and miR-155 as predictive biomarkers for the efficacy of chemoradiation in locally advanced head and neck squamous cell carcinoma. Eur J Cancer 2017, 77, 3-12, doi:10.1016/j.ejca.2017.02.018.
3. Zhao, X.; Zhang, W.; Ji, W. YB-1 promotes laryngeal squamous cell carcinoma progression by inducing miR-155 expression via c-Myb. Future Oncol 2018, 14, 1579-1589, doi:10.2217/fon-2018-0058.
4. Baba, O.; Hasegawa, S.; Nagai, H.; Uchida, F.; Yamatoji, M.; Kanno, N.I.; Yamagata, K.; Sakai, S.; Yanagawa, T.; Bukawa, H. MicroRNA-155-5p is associated with oral squamous cell carcinoma metastasis and poor prognosis. Journal of Oral Pathology & Medicine 2016, 45, 248-255, doi:https://doi.org/10.1111/jop.12351.
5. Shi, L.-J.; Zhang, C.-Y.; Zhou, Z.-T.; Ma, J.-Y.; Liu, Y.; Bao, Z.-X.; Jiang, W.-W. MicroRNA-155 in oral squamous cell carcinoma: Overexpression, localization, and prognostic potential. Head & Neck 2015, 37, 970-976, doi:https://doi.org/10.1002/hed.23700.
6. Kim, H.; Yang, J.M.; Ahn, S.H.; Jeong, W.J.; Chung, J.H.; Paik, J.H. Potential Oncogenic Role and Prognostic Implication of MicroRNA-155-5p in Oral Squamous Cell Carcinoma. Anticancer Res 2018, 38, 5193-5200, doi:10.21873/anticanres.12842.
7. Bersani, C.; Mints, M.; Tertipis, N.; Haeggblom, L.; Näsman, A.; Romanitan, M.; Dalianis, T.; Ramqvist, T. MicroRNA-155, -185 and -193b as biomarkers in human papillomavirus positive and negative tonsillar and base of tongue squamous cell carcinoma. Oral Oncol 2018, 82, 8-16, doi:10.1016/j.oraloncology.2018.04.021.
8. Wu, M.; Duan, Q.; Liu, X.; Zhang, P.; Fu, Y.; Zhang, Z.; Liu, L.; Cheng, J.; Jiang, H. MiR-155-5p promotes oral cancer progression by targeting chromatin remodeling gene ARID2. Biomedicine & Pharmacotherapy 2020, 122, 109696, doi:https://doi.org/10.1016/j.biopha.2019.109696.
